# Research on Through-Flame Imaging Using Mid-Wave Infrared Camera Based on Flame Filter

**DOI:** 10.3390/s24206696

**Published:** 2024-10-18

**Authors:** Fengxun Zheng, Guodong Sun, Yanpeng Suo, Hao Ma, Tengxiao Feng

**Affiliations:** 1 College of Agricultural Equipment Engineering, Henan University of Science and Technology, Luoyang 471009, China; 2 Luoyang Thermal Sense Technology Co., Ltd., Luoyang 471009, China

**Keywords:** through-flame imaging, thermal imager, MWIR, flame filter

## Abstract

High-temperature furnaces and coal-fired boilers are widely employed in the petrochemical and metal-smelting sectors. Over time, the deterioration, corrosion, and wear of pipelines can lead to equipment malfunctions and safety incidents. Nevertheless, effective real-time monitoring of equipment conditions remains insufficient, primarily due to the interference caused by flames generated from fuel combustion. To address this issue, in this study, a through-flame infrared imager is developed based on the mid-wave infrared (MWIR) radiation characteristics of the flame. The imager incorporates a narrowband filter that operates within the wavelength range of 3.80 μm to 4.05 μm, which is integrated into conventional thermal imagers to perform flame filtering. This configuration enables the radiation from the background to pass through the flame and reach the detector, thereby allowing the infrared imager to visualize objects obscured by the flame and measure their temperatures directly. Our experimental findings indicate that the imager is capable of through-flame imaging; specifically, when the temperature of the target exceeds 50 °C, the imager can effectively penetrate the outer flame of an alcohol lamp and distinctly capture the target’s outline. Importantly, as the temperature of the target increases, the clarity of the target’s contour in the images improves. The MWIR through-flame imager presents considerable potential for the real-time monitoring and preventive maintenance of high-temperature furnaces and similar equipment, such as detecting the degradation of refractory materials and damage to pipelines.

## 1. Introduction

High-temperature furnaces, gas furnaces, and coal-fired boilers are commonly utilized in industries such as petrochemicals and metal smelting. Over time, damage to the pipes inside the furnace walls can lead to equipment failure. As faults typically arise during operation, it is essential to monitor and detect the equipment’s condition in real-time. However, the high-temperature flames produced by fuel combustion can obscure the targets located behind and around them, making it challenging for conventional monitoring techniques to effectively capture images of these targets.

Currently, research on through-flame imaging is mainly focused on the blue region of the visible-light spectrum. This is because the spectral radiation of the flame in blue light (450 nm) is only one five-hundredth of the intensity of the flame in red light (750 nm) [1]. By adding a filter, which is only sensitive to shorter visible-light wavelengths, the camera may be able to “see” the target without being affected by the flames [2]. Early research improved the visibility of targets behind flames by using blue-light filters to block the strong radiation emitted by the flames. However, this method is not suitable for high-temperature flames [3,4]. Following these findings, subsequent studies aimed to improve through-flame imaging by using an active light source to compensate for target emissions [5], for instance, the addition of a 450 nm blue light source [6], as well as the use of a 400 nm violet light source [7]. Additionally, Zepper et al. use a copper vapor laser (CVL) working at 500 nm to obtain an image through aluminum flames [8]. Although the through-flame method based on visible light is cost-effective, it is still not practical due to the limited range of active light sources and the susceptibility of this band to smoke scattering [9].

Another optional approach for through-flame methods is to reconstruct the target behind the flame based on holographic interferometric imaging [10]. Mitchell et al. obtain three-dimensional images of objects obscured by methane or acetylene flames and track their deformation by employing coherent lasers [11]. Hoehler et al. employ a blue-laser sensor to measure the displacement of targets situated behind natural gas flames [12]. Locatelli et al. visualize individuals situated behind smoke and flames, based on 10.6 μm far-infrared digital holography, demonstrating that digital holography is capable of detecting individuals in motion through smoke and flames [13]. Subsequently, Chai et al. use a 10.6 μm CO_2_ laser and a long-wave uncooled infrared camera to obtain a holographic image of a target behind flames [14]. These imaging methods based on holography require a sophisticated optical system and remain in the experimental phase. Additionally, a long-wave laser light source poses a potential risk of eye injury. Other technologies, such as polarization imaging, are mainly used in atmospheric remote sensing and fog penetration. There is no published research on through-flame detection. Deep learning is also explored for object detection behind flames, but the elimination of flames remains a challenge [15].

Since a flame emits continuous mid-wave and long-wave infrared radiation, research on the infrared imaging of flames has focused on detecting the flame itself [16]. A flame detection system based on a mid-wave infrared imager has very significant flame detection capability [17]. However, through-flame imaging is fundamentally different from flame detection, as it requires minimizing the influence of the flame and choosing a wavelength band with reduced flame radiation for imaging. Currently, there is no literature available on through-flame imaging using mid-wave infrared technology. A report from the FLIR corporation in the United States described the GF309 through-flame thermal imager, which is a built-in flame filter for through-flame imaging and temperature measurement. This camera is used for detecting combustion furnaces and has a temperature measurement range of −40 °C to 1500 °C, but specific implementation details are not provided.

In response to this, we conducted a through-flame imaging experiment based on a mid-wave infrared thermal imager, in conjunction with a narrowband filter. The purpose of this research is to assess the capabilities of the MWIR imager in through-flame scenarios and to explore the potential applications of such thermal imaging technology.

## 2. Spectral Properties of Flame

Fuels can be categorized into solid, liquid, and gaseous fuels based on their physical state. Solid fuels encompass materials such as coal, firewood, and oil shale. Liquid fuels comprise petroleum derivatives, including gasoline, diesel, and heavy oil, in addition to ethanol. Gaseous fuels consist of natural gas, coal gas, liquefied gas, and acetylene, among others. The process of combustion is inherently complex, with the principal constituents of the flame during typical fuel combustion being carbon dioxide (CO_2_), carbon monoxide (CO), and water vapor (H_2_O) [18]. Certain flames, such as those produced by pulverized coal, may not achieve complete combustion, resulting in the presence of a significant quantity of solid particulates. Additionally, various intermediate species, including hydroxyl radicals (OH), cyanide (CN), methyl radicals (CH), and dicarbon (C_2_), can be found within the flame. In regions of elevated flame temperature, matter emits characteristic radiation, while in an area where the temperature is lower, it absorbs radiation from hotter regions. Furthermore, solid particles within the flame contribute to the reflection and scattering of radiation.

The spectral characteristics of flame radiation exhibit variations contingent upon the type of fuel utilized. Specifically, the peak spectral emissions of low-carbon compounds are predominantly observed within the ranges of 2.65–2.80 μm, 4.15–4.45 μm, 5.60–7.60 μm, and 13–17 μm, which correspond to the molecular absorption of water vapor and carbon dioxide. Generally, the combustion of hydrocarbon compounds primarily yields water vapor (H_2_O) and carbon dioxide (CO_2_) gasses [19]. For oil combustion, spectral peaks are identified at 2.40 μm, 2.80 μm, 4.20 μm, 4.50 μm, and 6.30 μm, with an additional spectral peak associated with the C-H bond at 3.40 μm [20]. In the case of alcohol lamp flames, the radiation spectrum exhibits peaks at 2.70 μm and 4.30 μm. The n-heptane flame displays spectral peaks at 2.10 μm, 2.70 μm, and 4.30 μm. Furthermore, the flame spectral emissions of carbon disulfide, along with various fuels, such as styrene, acetonitrile, and ethyl acetate, are primarily attributed to the molecular radiation of sulfur dioxide (SO_2_), water vapor, and carbon dioxide at high temperatures. The characteristic peaks for SO_2_ are noted to be 4.05 μm, 7.40 μm, and 8.51 μm. The spectral characteristics of acetonitrile and ethyl acetate combustion flames are largely similar, whereas the styrene flame exhibits significant carbon black radiation in addition to high-temperature gas emissions, with a central wavelength of 7 μm. The spectral characteristics of wood and candles exhibit a nearly horizontal linear profile within the wavelength range of 3.30 to 4.10 μm, lacking discernible features. Furthermore, research conducted by Chen et al. has examined the spectral properties of decoy bomb flames, focusing on the influence of various additives on flame radiation [21]. Wang et al. performed an analysis of the flame spectra associated with two nitrogen-containing compounds, acrylonitrile and acetonitrile, as well as anhydrous ethanol, under controlled indoor conditions [22].

**Figure 1:**
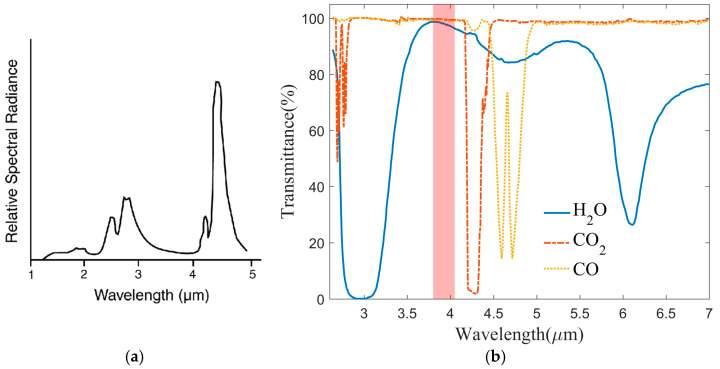
(**a**) Infrared emission spectrum of a Bunsen burner flame [23]; (**b**) transmittance of CO_2_, CO, and H_2_O gasses.

Integrating these studies indicates that the flame spectrum predominantly arises from the molecular radiation of H_2_O and CO_2_ present in the combustion byproducts. Figure 1a, cited from the work of Underhill-Shanks K. et al. [23], shows an infrared spectrum obtained from a Bunsen burner. Both the H_2_O and CO_2_ bands are present in this figure. Figure 1b illustrates the infrared spectrum of several gas molecules within the mid-infrared wavelength range. The data come from the HITRAN2020 molecular spectroscopic database [24]. From Figure 1b, it can be seen that the high transmittance is aligned with the low spectral radiation shown in Figure 1a. Notably, Figure 1 indicates a radiation window, marked with a red column, where there is minimal absorption of H_2_O and CO_2_ gasses within this specific wavelength range.

## 3. MWIR Through-Flame Imaging

### 3.1. Principle of Through-Flame Imaging

The flame demonstrates two high-transmittance regions within the spectrum ranging from visible to infrared light, similar to the atmospheric window, specifically located in the blue-light region (near 400 nm) and the mid-wave infrared region (near 3.90 µm) [18]. These two wavebands are applicable to through-flame imaging. Cameras that operate in visible light mainly depend on reflected light for imaging, which makes them susceptible to ambient lighting conditions. At the same time, they are also vulnerable to interference from glow and smoke, due to the short wavelength of visible light. In contrast, infrared detectors utilize thermal radiation emitted by the target itself, exhibiting heightened sensitivity to high-temperature objects within the scene. The standard wide-band infrared thermal imagers can capture all thermal radiation within their designated spectral response range, encompassing both the self-emitted radiation of flames and the radiation from the background. The process can be described as follows:

(1) $$I\left(\lambda \right)={\rho_{t}\left(\lambda \right)I}_{target}\left(\lambda ,T_{1}\right)+I_{flame}\left(\lambda ,T_{2}\right),$$

where I represents radiation intensity, and $\rho_{t}\left(\lambda \right)$ represents the transmittance of the flame at the given wavelength. Obviously, a critical aspect of mid-wave through-flame imaging lies in minimizing the influence of flame radiation on background heat radiation.

The flame can be regarded as a gray body with an emissivity of ε; then, the second item on the left side of the above equation can be described as follows:

(2) $$I_{flame}\left(\lambda \right)={\varepsilon I}_{b}\left(\lambda ,T_{2}\right),$$

where $I_{b}$ represents the radiation of a blackbody with a temperature equal to $T_{2}$, which can be calculated by Planck’s law. According to the conservation of energy, the sum of an object’s transmittance, absorptance, and reflectance is equal to 1. Since gasses hardly reflect thermal radiation, we have $\rho_{t}\left(\lambda \right)=1-\rho_{a}\left(\lambda \right)$, where $\rho_{a}\left(\lambda \right)$ represents the absorptance. According to Kirchhoff’s law, under thermal equilibrium conditions, the emissivity of a flame is numerically equal to its absorptivity. Consequently, Equation (1) can be rewritten as follows:

(3) $$I\left(\lambda \right)={\rho_{t}\left(\lambda \right)I}_{target}\left(\lambda ,T_{1}\right)+\left[1-\rho_{t}\left(\lambda \right)\right]I_{b}\left(\lambda ,T_{2}\right),$$

**Figure 2:**
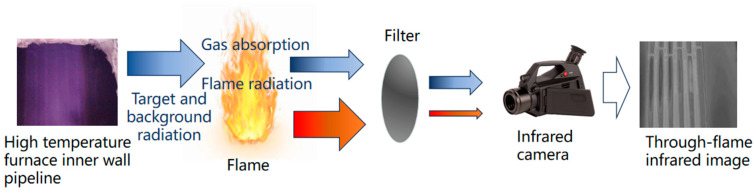
A schematic diagram of MWIR through-flame imaging.

**Figure 3:**
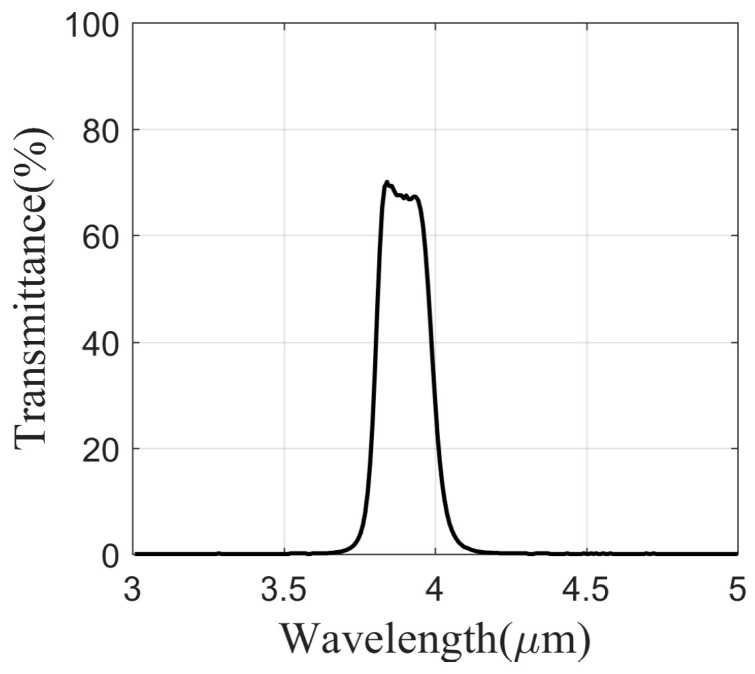
The transmittance curve of the flame filter.

From Equation (3), transmittance is an important parameter that affects through-flame imaging, and choosing appropriate infrared wavebands may reduce the impact of the flame. Based on this, the fundamental principles governing MWIR through-flame imaging are illustrated in Figure 2. The radiation energy that traverses the flame comprises both the flame’s radiation and the background radiation that has been attenuated by the flame. Filters can be employed to eliminate the flame radiation, thereby enabling the infrared camera to detect the energy of the background radiation. The formula for calculating the radiant intensity after passing through the filter is as follows:

(4) $$I=\int_{\lambda_{1}}^{\lambda_{2}}I\left(\lambda \right)d\lambda ,$$

where $\lambda_{1}$ and $\lambda_{2}$ are the cut-off wavelengths of the filter. As shown in Figure 1, the weak absorption band near 3.90 μm, which corresponds to the low-radiation band, is highly effective for detecting targets hidden by flames. In the process of selecting the filter range, it is essential to maximize the filter width while simultaneously excluding the primary radiation of the flame. This approach optimizes the utilization of radiation energy and enhances the signal-to-noise ratio in imaging. In the present study, the filter bandwidth was established to be between 3.80 and 4.05 μm, effectively circumventing the absorption peaks associated with water vapor, C-H bonds, and SO_2_ gas present in the flame. Figure 3 shows the transmittance curve of the flame filter, and the performance details are listed in Table 1. The center wavelength is located at 3.92 μm, with a peak transmittance of 70%. The selection of a lower transmittance is primarily aimed at diminishing the intensity of incident radiation; thereby, the imager is suitable for measuring high-temperature objects.

### 3.2. The Through-Flame Thermal Imager

**Figure 4:**
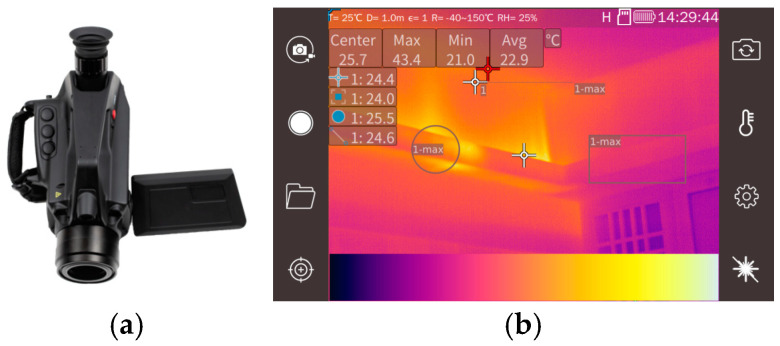
The FG390 camera (**a**) and its infrared image with temperature measurement (**b**).

The thermal imager used in this study was the FG390 model, which was developed by Luoyang Thermal Technology Co., Ltd., a subsidiary of Beijing Fujirui Optoelectronics. The company is located in Luoyang City, Henan Province, China. The FG390, depicted in the left image of Figure 4, is designed for remote, real-time safety assessments of various equipment, including gas stoves, chemical heaters, and coal-fired boilers. The device is capable of through-flame imaging and temperature measurement. It is equipped with a 320 × 256-pixel indium antimonide (InSb) focal-plane array (FPA) detector, featuring a pixel pitch of 30 μm, which is cyclically cooled by a Stirling chiller, achieving a thermal sensitivity of less than 15 mK. Additionally, the thermal imager incorporates a built-in filter with a wavelength range of 3.80 to 4.05 μm positioned in front of the detector focal plane. The FG390 also offers multi-point temperature measurement capability, allowing for the addition of temperature measurement points or areas. The temperature measurement range spans from −20 °C to +1500 °C, and it can produce pseudo-color infrared images, as illustrated in the right image of Figure 4. The device provides data on the center point temperature, as well as the maximum, minimum, and average temperatures of the observed scene.

## 4. Experiment and Results of Through-Flame Imaging

### 4.1. Through-Flame Imaging Experiment

**Figure 5:**
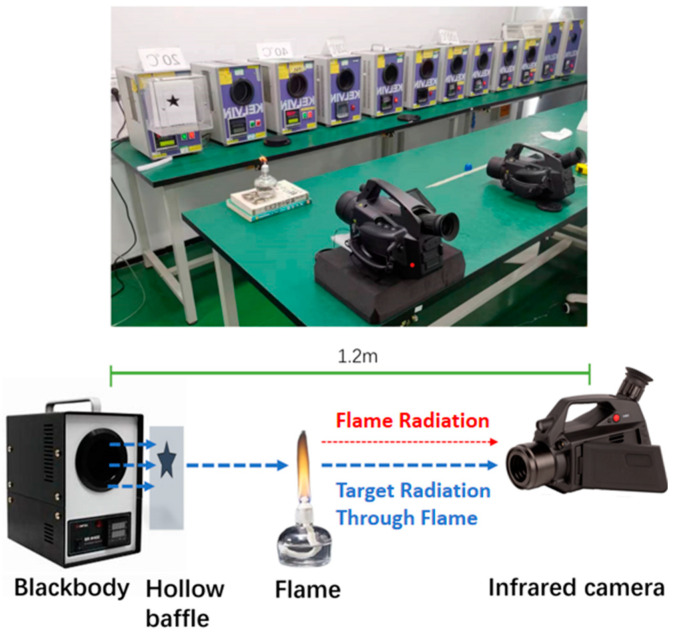
The experimental composition of MWIR through-flame imaging.

The effect of MWIR through-flame imaging is influenced by several variables, including the temperatures of the target, the flame, and the background, the detection distance, as well as the particulate matter within the flame. In the case of pure flames, the flame and target temperature are the primary factors affecting the through-flame effect. Despite the capability of through-flame imaging to circumvent the peak radiation zone of the flame, the high temperature of the flame can still obscure the radiation from a low-temperature background, thereby complicating the penetration of the flame. In this research, we performed an experiment utilizing the FG390 MWIR imager. The experimental setup involved a blackbody and a hollow board serving as detection targets, with a flame produced by an alcohol lamp. The thermal imager and the targets were aligned at the same height, and the thermal imager was situated 1.20 m away from the targets. The configuration and scene of the experiment are illustrated in Figure 5. The background temperature plays a crucial role in determining the infrared radiation intensity of the targets. During the experiment, we varied the temperature of the blackbody from 20 °C to 300 °C, selecting eight distinct temperature points for analysis. Additionally, a standard visible-light camera and a mid-wave cooled (wide-band imaging) camera were employed for comparative purposes.

**Figure 6:**
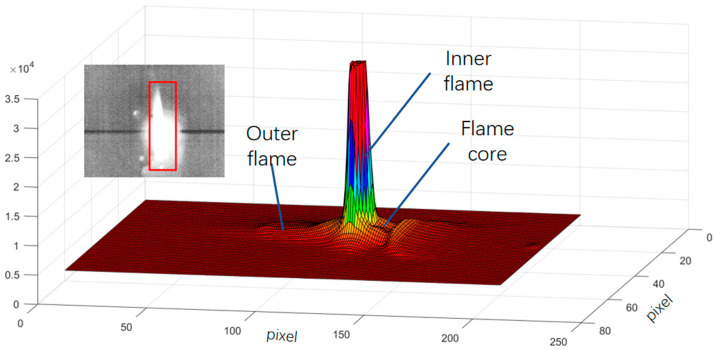
A 3D view of the alcohol lamp flame.

The flame produced by the alcohol lamp can be categorized into three distinct regions: the flame core, the inner flame, and the outer flame. Among these, the inner flame exhibits the highest temperature, while the flame core has the lowest temperature. During the experimental procedure, the flame height was measured to be between 4 and 5 cm, with the temperature of the inner flame ranging from approximately 400 to 500 °C. Additionally, the outer flame displayed a slight oscillation of 1 to 2 cm, attributed to disturbances in the indoor air environment. The outer flame emits a color of orange-yellow within the visible-light spectrum; therefore, in this investigation, the target was positioned behind the outer flame at an appropriate imaging angle to facilitate a comparison with visible light. Figure 6 illustrates the three-dimensional distribution of the infrared raw data following the application of a flame filter, revealing a significant reduction in the energy of the outer flame post-filtering. The raw data refer to the 14-bit digital number (DN) values that have undergone non-uniformity correction and temperature calibration but have not yet been converted into 8-bit image data.

### 4.2. Results of Experiment

#### 4.2.1. Comparison of Visible and Infrared Images

**Figure 7:**
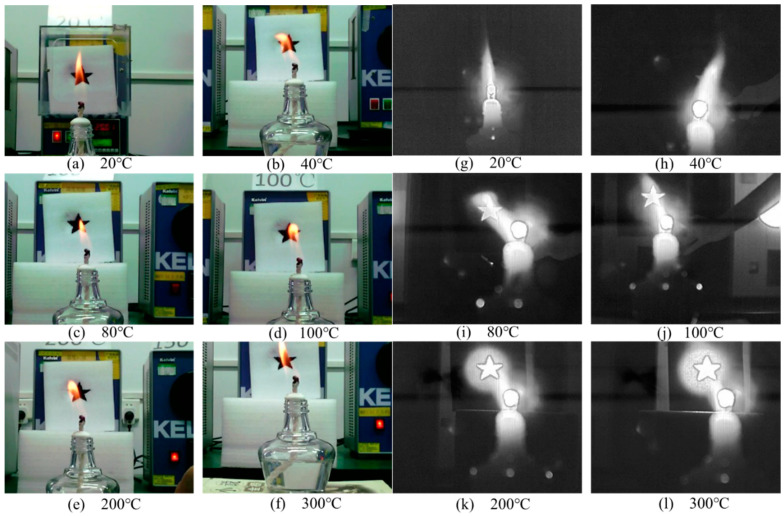
The visible-light images (**a**–**f**) and infrared images (**g**–**l**) at different target temperatures of 20 °C to 300 °C.

Figure 7 presents six sets of images, comprising ordinary visible-light images and infrared through-flame images, corresponding to various target temperatures. As illustrated in Figure 7a–f, the flame core temperature of the alcohol lamp is relatively low, resulting in a transparent appearance that does not significantly impact the visible-light image. The visible light that obscures the target primarily emanates from the orange-yellow outer flame, which completely obstructs the target located behind it, rendering the target’s outline indistinguishable. Notably, as the target temperature increases, there is no marked alteration in the visible-light image, suggesting that the flame’s occlusion is independent of the target’s temperature when the target itself does not emit light. From Figure 7g–l, it is observed that at target temperatures of 20 °C and 40 °C, the target remains entirely obscured by the flames and is thus unobservable. At a target temperature of 80 °C, the infrared radiation emitted by the target is largely unaffected by the flames, allowing for the outline of the object to become visible. As the target temperature continues to rise, the clarity and completeness of the target’s outline improve. The obscuration of the target due to the flame’s radiation is evident in Figure 7k–l where the high temperature facilitates the passage of surface source blackbody radiation through the hollow board, resulting in a circular background. The shape of the flame remains indistinguishable within the temperature range of 200 °C to 300 °C.

#### 4.2.2. Comparison of Wide-Band and Through-Flame Images

**Figure 8:**
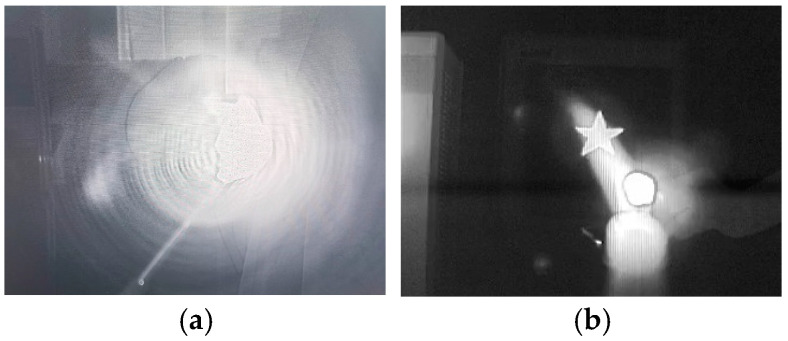
Comparison of wide-band infrared image (**a**) and through-flame image (**b**).

The wide-band thermal imagers capture higher radiation energy compared to the through-flame thermal imagers equipped with a flame filter. Thus, the data obtained from wide-band thermal imagers are prone to saturation when hot objects are present. In the conducted experiment, the integration time of the wide-band thermal imager was minimized, and the uniformity coefficient was calibrated using a two-point correction method. Figure 8a illustrates the imaging results from the wide-band thermal imager. As can be seen from the figure, the intense temperature of the flame causes the energy radiation from the flame to dominate the image, obscuring the target located behind it. Conversely, Figure 8b depicts the through-flame image, which, after applying flame filtering, can discern both the flame and the target situated behind it more effectively.

#### 4.2.3. Influence of Target Temperature on Through-Flame

**Figure 9:**
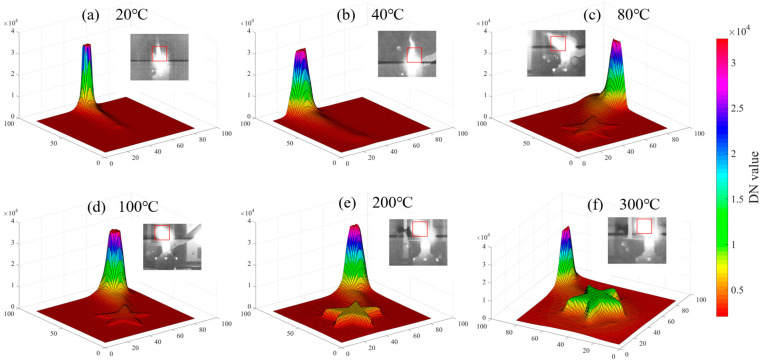
Six 3D views at different temperature levels, illustrating the variations in the raw data (digital number value) across the 80 × 80 pixel area of the alcohol lamp’s outer flame and the target.

Figure 9 illustrates a 3D representation of the raw data values within an 80 × 80 pixel area surrounding the target at various temperatures. The mean values (the average of the 10 × 10 pixel area encompassing the target) corresponding to the six distinct target temperatures are 2225, 2113, 3510, 3445, 6420, and 11,354, respectively. To facilitate the comparison, the mean DN value of the outer flame was computed. Due to the fluctuations in the flame, continuous multi-frame averaging was applied to the outer flame region, yielding an average value of 2153. This mean value is comparable to those of the targets at 20 °C and 40 °C, which complicates the differentiation of the target from the flame in the thermal image. With a increase in the target temperature, as shown in Figure 9c–f, the DN value of the target becomes progressively less influenced by the flame. According to the principle of through-flame thermal imaging, it is observed that while CO_2_ and H_2_O present in the flame exhibit minimal radiation and absorption within the waveband from 3.80 to 4.05 μm, the flame nonetheless emits a certain level of fundamental radiation in this band. Consequently, for MWIR through-flame imaging, a sufficient background temperature is necessary.

**Figure 10:**
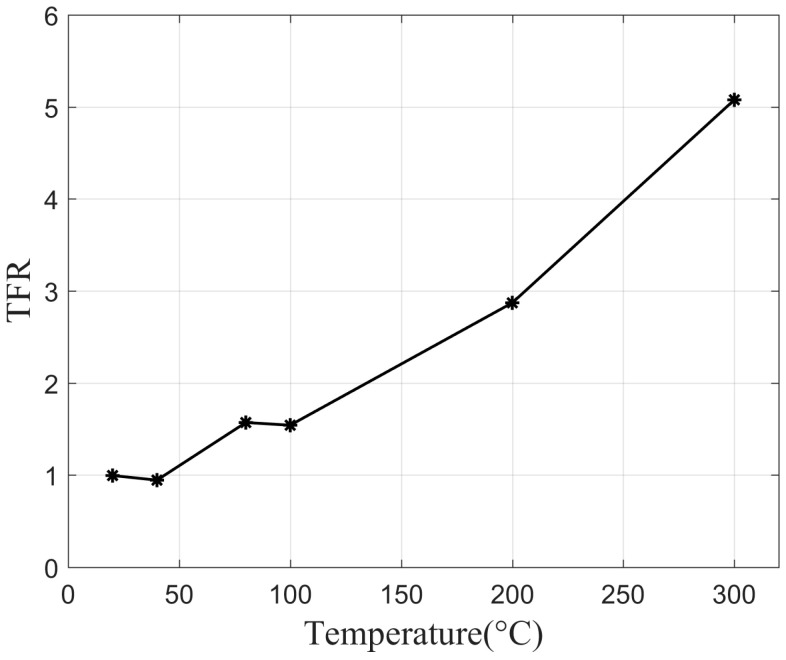
Curve of target–flame ratio (TFR) versus target temperature.

Furthermore, the target–flame ratio (TFR) is defined to intuitively represent the impact of the target’s temperature on penetrating the flame.

(5) $$TFR=\frac{I}{I_{flame}}=\frac{{DN}_{t}}{{DN}_{f}},$$

where ${DN}_{t}$ and ${DN}_{f}$ represents the average gray values of the targets and outer flame, respectively. When TFR is more than 1, the radiation of the flame will not easily be able to mask the radiation from the target. The higher the value of TFR, the greater the difference in the image, which indicates that the through-flame imaging is more effective. The TFR at six selected temperatures is shown in Figure 10. The inflection points in Figure 10 represent the TFR values at six selected temperatures, respectively. The TFR value increases significantly with the target temperature. When the temperature is below 50 degrees Celsius, the TFR value is around 1, indicating that the radiation of the background and the flame is comparable at this temperature. When the background temperature rises to 200 degrees Celsius, the radiation intensity of the target detected through the flame is 2.9 times that of the flame radiation. As the temperature rises to 300 degrees Celsius, the difference in radiation intensity approaches 5.1 times.

To clarify the specific target temperature, which can be visualized through the alcohol lamp, the blackbody was set to temperatures ranging from 40 °C to 80 °C, and it was observed that the contours of the targets with temperatures greater than 50 °C could be more effectively discerned in the continuous imaging. It is essential to recognize that this temperature threshold may change under different settings or environments, such as when modifying the detector’s integration time or the flame’s temperature. In other words, the result of 50 °C was the result for the specific experimental conditions and imager settings used. As the temperature of the flame rises, the target temperature must be correspondingly elevated to achieve a consistent through-flame effect. Concurrently, the thermal imager must adjust to the appropriate correction parameters, such as the nonuniformity correction coefficient, and an additional attenuator is also necessary, especially when the flame temperature is more than 800 °C.

### 4.3. Tests in Actual Environments

To verify the actual effect of the proposed through-flame imager, we conducted tests on a tube furnace at a petrochemical facility. The tube furnace is a crucial component of the tar distillation system, responsible for heating dehydrated tar, which is pumped by a tar pump, to temperatures between 380 and 405 °C. This process allows for the separation of light oil and phenol oil from coal tar. However, variations in the quality of the tar and raw materials can result in coking within the tube furnace, which may affect the operational safety of tube furnaces. The furnace uses a mixture of methane and hydrogen gas, which burn in the center, generating a flame temperature of around 1000 °C. The oil-pumping pipelines are arranged around the inner wall, and there is an observation window on one side of the furnace for internal inspection. From this window, the tar flow tube is positioned behind the flame.

**Figure 11:**
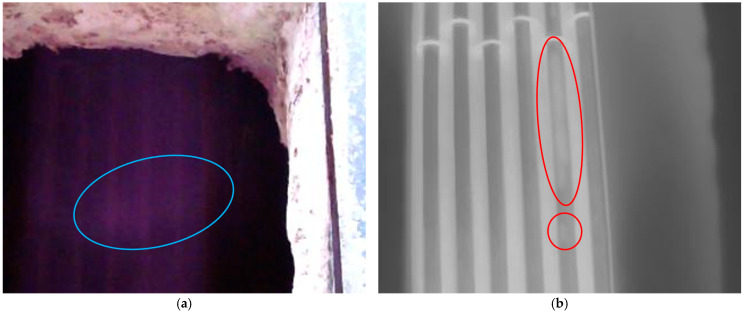
A visible-light image (**a**) and an infrared image (**b**) of pipelines in a tube furnace captured by the through-flame imager.

Figure 11 displays the visible-light image (a) and infrared image (b) of the tubes captured by the through-flame imager. In the visible-light video, a flickering flame is clearly visible, indicated by a blue circle in Figure 11a, while the pipeline behind the flame is nearly obscured. The infrared image eliminates the flame’s interference, allowing for a clear view of the pipeline contours along the furnace wall. The dark vertical bar in Figure 11b represents the oil tube, with the tar flowing inside it resulting in a lower temperature compared to the furnace wall. Notably, Figure 11b reveals coking occurring inside the first furnace tube on the right, as indicated by the red circle in Figure 11b. Coking increases the resistance to tar flow, raises the outlet pressure of the tar pump, and decreases the thermal conductivity of the tubes. Consequently, the temperature of the tubes rises, leading to increased surface oxidation and corrosion, which can thin the tube walls. Temperature measurements indicate that the area marked with a red circle reaches 474 degrees Celsius, which exceeds the normal temperature for furnace tubes. In extreme cases, this may cause the tubes to rupture, resulting in accidents.

Even though the actual temperatures of the flame and target differ from the temperature settings of the alcohol lamp and blackbody in controlled indoor conditions, they yield similar results when observed through the flame. By using the chosen mid-wave band, the radiation emitted by the target, which is much cooler than the flame, can pass through the flame and reach the detector, enabling the imager to capture the outline of the target located behind the flame.

## 5. Discussion

Through-flame imaging presents significant challenges, particularly due to the difficulties associated with eliminating flame interference for a conventional visible-light camera. MWIR imaging that uses a weak flame radiation band (3.80–4.05 um) exhibits significant potential for application in through-flame imaging. The primary purpose of this research is to assess the practicality of such technology implemented using a narrowband filter for through-flame imaging. The influence of target temperature on the through-flame images is also analyzed in an ideal laboratory environment. In our study, the pure alcohol lamp flames and simple geometric targets simulated by the blackbody are used during the tests, which still have significant gaps in real application scenarios. Thus, further in-depth research still needs to be conducted.

Our results suggest that the selected band is suitable for through-flame imaging, with the target temperature being the primary factor influencing the imaging effect. However, it should be noted that the outer flame of the alcohol lamp used in the experiment had a relatively low temperature, and the flame temperature was not included as a variable for analysis. It is evident that flame temperature, which can vary from several hundreds to thousands of degrees depending on different combustion substances, also plays a crucial role in affecting through-flame imaging. Therefore, experiments involving flames with different temperatures, particularly those with higher temperatures, are the primary consideration for future research. Furthermore, while the proposed waveband from 3.80 to 4.05 μm is suitable for most flames based on spectral analysis of various flames, significant differences in flame morphology and composition, due to different combustion conditions, cannot be overlooked. The imaging effect also requires further validation under different combustible conditions. Additionally, this study does not address the through-flame temperature measurement capability provided by the proposed infrared imager; therefore, assessing its accuracy in measuring temperatures using this method represents an important direction for future research efforts.

This through-flame research has two main applications: safety detection in power and industrial fields, such as high-temperature furnaces and coal-fired boilers, and rescue at the scene of a fire. These applications differ significantly. The former takes place in a controlled environment with known combustibles and flame temperatures. The imaging target is the inner wall of the furnace, which consistently emits high temperatures and radiation, making it convenient for thermal imager applications. Optimization techniques, such as adjusting detector parameters, recalibrating temperature coefficients, and adding attenuation filters, can improve imaging quality. In contrast, fire scene rescue is often complex due to thick smoke from inadequate combustion in residential buildings, workshops, shopping malls, or other environments. Further research is needed to determine if flame penetration technology can be applied effectively in these scenarios. This remains a long-term and meaningful topic for exploration.

## 6. Conclusions

Based on the spectral radiation characteristics of the flame within the mid-wave infrared spectrum, this research was conducted using through-flame imaging in a controlled laboratory environment. The methodology incorporated a flame filter, operating within the wavelength range of 3.80 to 4.05 µm, to mitigate the interference resulting from the absorption and emission of carbon dioxide (CO_2_) and water vapor. Our primary findings are summarized as follows.

(1) The target obscured by the flame is not discernible through a standard visible-light camera, as the coloration of the flame in the image conceals the outline of the target. Furthermore, temperature fluctuations in the target do not affect this phenomenon. In contrast, a thermal imager equipped with a flame filter is unaffected by the flame’s color, allowing for clear visualization of the target’s contours.
(2) The ordinary MWIR imager receives all the thermal radiation in a wide band, including the strong radiation of CO_2_ and water vapor in the flame. It is easily saturated and unable to capture the radiation of the background target. The implementation of a flame filter effectively addresses this limitation, enabling the imaging of objects obscured by flames.
(3) The target temperature has a direct influence on the through-flame effect for thermal imaging. Our experimental findings utilizing the flame of an alcohol lamp indicate that when the target temperature surpasses 50 °C, the infrared thermal imager can penetrate the outer flame of the alcohol lamp, thereby allowing for clear visualization of the target’s contour. Furthermore, it is noted that an increase in the target temperature corresponds with a more defined outline of the target in the image behind the flame.

## Figures and Tables

**Table 1:** The performance details of the flame filter.

Parameter	Value
Center wavelength (CWL)	3.92 μm
Band width (BW)	205 nm
Peak transmittance (Tx Pk)	70.04%
Blocking	Fully blocked

## Data Availability

Data are contained within the article.
